# Prevalence and Correlates of Obsessive-Compulsive Symptoms in Adolescent Patients with Depressive and Anxiety Symptoms

**DOI:** 10.31083/AP38997

**Published:** 2025-03-13

**Authors:** Lingjun Chu, Nan Jiang, Xiwang Fan

**Affiliations:** ^1^Clinical Research Center for Mental Disorders, Shanghai Pudong New Area Mental Health Center, School of Medicine, Tongji University, 200124 Shanghai, China

**Keywords:** adolescents, depressive, obsessive-compulsive symptoms, comorbidity, drug-naive, first-episode

## Abstract

**Background:**

Obsessive-Compulsive Disorder (OCD) patients are often comorbid with depression and anxiety. However, limited research has explored this comorbidity from the perspective of individuals with depression and anxiety exhibiting obsessive-compulsive symptoms (OCS). This study aims to investigate the prevalence and potential associations between depression, anxiety, and OCS in the adolescent patient population.

**Methods:**

A retrospective study was employed in this research. A total of: 327 drug-naive, first-episode adolescent patients aged 10 to 19 years, presenting both depressive and anxiety symptoms, were recruited from the Shanghai Pudong New Area Mental Health Center in China. The Chinese version of the Symptom Checklist-90 (SCL-90) was used to assess the severity of OCS. Binary logistic regression was applied to analyze the influence of depression and anxiety levels on OCS.

**Results:**

More than half (52.3%) of the 327 adolescent participants with depressive and anxiety symptoms had severe obsessive-compulsive symptoms (OCS). Additionally, 35.9% had moderate OCS, 12.9% had mild OCS, and only 2.8% were symptom-free. The results also indicated a significant correlation between OCS and both depression (β = 0.073, Wald χ^2^ < 0.001, *p* < 0.005) and anxiety levels (β = 0.066, Wald χ^2^ < 0.005, *p* < 0.001).

**Conclusions:**

The findings provide valuable insights into the predictive ability of depression and anxiety level in the development of OCS and OCD during adolescence, highlighting the importance of early identification and intervention. Future studies should include a larger and more diverse sample, with the incorporation of professional clinical evaluations to further verify these results.

**Clinical Trial Registration:**

The study was registered at https://www.chictr.org.cn/, registration number: ChiCTR2300070007.

## 1. Introduction

Obsessive-Compulsive Disorder (OCD) is characterized by obsessions and/or compulsions. Obsessions are intrusive, unwanted thoughts, urges, or images, while compulsions are repetitive behaviors or mental acts performed in response to an obsession or according to rigid rules [1]. Obsessive-Compulsive Symptoms (OCS) refer to the presence of obsessive and/or compulsive symptoms that do not meet the diagnostic criteria for OCD but still significantly impact the individual’s daily functioning.

Depression, particularly Major Depressive Disorder (MDD), is a mental health disorder characterized by a persistent depressed mood or a marked loss of interest or pleasure in most activities, lasting for an extended period of time, typically two weeks or longer [1]. It could be viewed as a significant contributor to the global burden of disease and often manifests during adolescence [2]. Adolescence is a critical and unstable period marked by psychosocial, physiological, and cognitive changes that can make young people more susceptible to psychological disorders. Globally, 34% of adolescents aged 10 to 19 are at risk of developing clinical depression, with female adolescents at a higher risk [3]. Unlike adult depression, adolescent depression often presents with more irritability than sadness and frequently coexists with other psychiatric conditions, including anxiety and OCS [4, 5, 6]. Tiller (2013) [7] showed a high comorbidity rate between depression and anxiety; for example, 90% of patients with anxiety exhibit depressive symptoms, while 85% of those with depression experience severe anxiety.

OCD was historically classified under the umbrella of generalized anxiety disorder (GAD) in the DSM-IV due to shared features such as “intolerance of uncertainty” and the presence of repetitive, intrusive, and distressing thoughts [8]. However, OCD and GAD also have distinct characteristics [8]. Obsessions in OCD are more likely to involve unusual or bizarre themes and are often experienced as distressing or alien to a person’s sense of self, whereas worries in GAD typically focus on realistic, everyday concerns. Additionally, individuals with OCD tend to make greater efforts to control their thoughts compared to those with GAD. As a result, in the DSM-V, OCD was removed from the anxiety disorders category and placed in a new group called obsessive-compulsive and related disorders (OCRDs) [1]. Despite this reclassification, there remains a high comorbidity between OCD and anxiety disorder [8].

In addition to its comorbidity with anxiety, OCD is also frequently associated with depression [9, 10]. Research indicates that depressive symptoms are common in individuals with OCD, often as a consequence of the significant burden imposed by OCD on the individual’s quality of life and functioning across work, family, and social domains [11]. As posited by Goodwin (2015) [12], the overlap between OCD, depression, and anxiety may be illustrated by shared genetic and brain mechanisms [12]. For example, genetic variations in the serotonin transporter gene (SLC6A4) have been linked to all three conditions, suggesting a common genetic predisposition [12]. Additionally, neuroimaging studies have shown similar patterns of abnormal brain activity across these conditions, particularly in the amygdala, which plays a crucial role in emotion processing and fear conditioning [12]. This implies that not only can OCD patients have comorbid depressive or anxiety symptoms, but also individuals with depression or anxiety may exhibit symptoms of OCD. However, the existing literature primarily focuses on how OCD or OCS predicts depression and anxiety. For example, Hofmeijer-Sevink *et al*. (2018) [13] found that OCS is strongly associated with both anxiety and depressive disorders and may predict their prevalence and severity. However, there is limited research examining whether the inverse is true, that is, whether depression and anxiety can predict the onset or severity of OCS.

The current study seeks to address this gap by investigating whether adolescent patients with depressive symptoms comorbid with anxiety symptoms also exhibit OCS. Adolescents aged 10 to 19 are chosen for this study due to their heightened vulnerability to developing clinical depression [3]. Specifically, this research aims to examine: (1) whether adolescent patients with depressive symptoms comorbid with anxiety symptoms also exhibit OCS; (2) whether gender differences exist in the prevalence of OCS among adolescents with depressive symptoms comorbid with anxiety symptoms; and (3) whether the level of depressive symptoms and anxiety symptoms could individually predict OCS.

Based on the existing literature highlighting the high comorbidity among OCD, depression, and anxiety, the following hypotheses are proposed: (1) a substantial portion of adolescent patients with depression and anxiety symptoms will have comorbid OCS; (2) female adolescents with depressive symptoms comorbid with anxiety symptoms will be more likely to develop OCS compared to male adolescents; and (3) both depression and anxiety level could individually predict OCS.

## 2. Materials and Methods

### 2.1 Study Design and Settings

A retrospective study was used for the present research, and the data were collected from the Shanghai Pudong New Area Mental Health Center. All participants completed three self-report questionnaires at the Shanghai Pudong New Area Mental Health Center under the supervision of a clinical psychologist.

### 2.2 Participants

A total of 327 adolescent patients (female = 264, male = 63) aged 10–19 years were enrolled at the Shanghai Pudong New Area Mental Health Center from January 2021 to May 2023. Only patients experiencing both major depression and comorbid anxiety, identified as first-episode cases without prior exposure to medication, were included in the study.

This study received ethical approval from the Ethics Committee of the Shanghai Pudong New Area Mental Health Center in China (PDJW-IIT-2022-011GZ1) and was registered at https://www.chictr.org.cn/ (clinical trials registry number: [ChiCTR2300070007]). All participants provided written informed consent. The study was conducted in accordance with the Declaration of Helsinki.

### 2.3 Materials

In this study, Self-Rating Depression Scale (SDS), Self-Rating Anxiety Scale (SAS), and The Chinese Version of the Symptom Checklist-90 (SCL-90) scales were used to assess participants’ symptoms of depression, anxiety, and obsessive-compulsive disorder. These questionnaires are validated self-report tools designed to assess the severity of symptoms and are used to screen individuals with potential mental health issues [12, 13, 14].

#### 2.3.1 Self-Rating Depression Scale (SDS)

The SDS by Zung (1965) is a 20-item questionnaire that measures symptoms of depression [14]. Participants rate their feelings over the past week for each item on a scale from 1 to 4, with higher scores indicating more frequent symptoms. The SDS raw score ranges from 20 to 80, but it is typically converted to the SDS Index on a 100-point scale for presentation. According to the Chinese SDS manual, scores between 50 and 59 indicate mild depression, scores between 60 and 69 indicate moderate depression, and scores of 70 or above indicate severe depression.

#### 2.3.2 Self-Rating Anxiety Scale (SAS)

The Self-Report Anxiety Scale (SAS) by Zung (1971) consists of 20 items, each rated on a 4-point Likert scale, covering cognitive, autonomic, motor, and central nervous system symptoms of anxiety [15]. The total score, ranging from 20 to 80, is converted to an anxiety index score from 25 to 100, with higher scores indicating greater anxiety severity.

#### 2.3.3 The Chinese Version of the Symptom Checklist-90 (SCL-90)

In this study, the Chinese version of the Symptom Checklist-90 (SCL-90) was used to assess OCS in patients. SCL-90 is a self-report tool for assessing mental health disorder symptoms. The Chinese version of SCL-90 was validated by Wang *et al*. (1999) [16], who established normative values for different symptoms among adolescents by studying 5849 Chinese students from junior and senior high schools. Their research revealed that the average score for OCS among Chinese adolescents was 1.80. The Chinese version of SCL-90 questionnaire comprised 90 items, each rated on a five-point Likert scale from 1 (not at all) to 5 (extremely). Higher scores on the Chinese version of SCL-90 indicated more severe psychological problems experienced in the past week. The severity of specific psychological issues was indicated by individual subscale scores. Individuals with a standardized subscale score (the sum of all subscale scores divided by the number of items) ≥3 were identified as having moderate to severe specific psychological problems. Consequently, in the present study, a score exceeding 3 on the SCL-90 for OCS was employed as the threshold for identifying patients with OCS. According to previous studies, the Chinese version of SCL-90 was demonstrated to have good psychometric properties with satisfactory reliability and validity [15, 16].

### 2.4 Statistical Analysis

Data analysis was performed using Python (Version 3.10.1, Python Software Foundation, Wilmington, DE, USA) and Visual Studio Code (Version 1.94.2, Microsoft, Redmond, WA, USA), utilizing the Chi-Square Test to examine demographic data and assess the prevalence of OCS in adolescents with comorbid depressive disorder and anxiety. Binary logistic regression was employed for further analysis.

## 3. Results

### 3.1 Prevalence of OCS Comorbidity in Adolescent Patients with Depressive and Anxiety Symptoms

#### 3.1.1 Prevalence of Depressive and Anxiety Symptoms

The prevalence of depressive symptoms among the 327 adolescent participants, as assessed by the Self-Rating Depression Scale (SDS), was 11.6% with mild depression (n = 38), 31.7% with moderate depression (n = 104), and 56.4% with severe depression (n = 185). These findings highlight the significant presence of moderate-to-severe depression in this population.

The prevalence of anxiety symptoms among the 327 adolescent participants, as assessed by the Self-Rating Anxiety Scale (SDS), was 41.2% with mild anxiety (n = 135), 33.2% with moderate anxiety (n = 109), and 25.3% with severe anxiety (n = 83). These findings indicate that the entire patient participant population is experiencing various levels of anxiety, with mild anxiety being the most common.

#### 3.1.2 Prevalence of Obsessive-Compulsive Symptoms

Table 1 presents the prevalence of OCS among the adolescent participants, categorized by age, gender, depression, and anxiety levels. The data demonstrated significant variations in OCS prevalence across anxiety and depression levels.

**Table 1.** **Prevalence of obsessive-compulsive symptoms in adolescents with comorbid depressive disorder and anxiety**.

Variable		Obsessive-compulsive level				χ ^2^	*p*
		Healthy	Mild	Moderate	Severe		
Count, n, %		9 (0.028)	39 (0.119)	108 (0.33)	171 (0.523)	981.000	<0.001
Sex (n, %)	Female	5 (0.02)	28 (0.11)	84 (0.32)	147 (0.56)	9.287	0.026
	Male	4 (0.06)	11 (0.17)	24 (0.38)	24 (0.38)		
Age (n, %)	Early adolescence (10–13 years)	0 (0.0)	6 (0.19)	13 (0.41)	13 (0.41)	7.594	0.269
	Mid-adolescence (14–17 years)	7 (0.03)	25 (0.11)	67 (0.3)	128 (0.56)		
	Late adolescence (18–19 years)	2 (0.03)	8 (0.12)	28 (0.41)	30 (0.44)		
Depression level (n, %)	Mild	4 (0.11)	13 (0.34)	11 (0.29)	10 (0.26)	68.174	<0.001
	Moderate	4 (0.04)	19 (0.18)	46 (0.44)	35 (0.34)		
	Severe	1 (0.01)	7 (0.04)	51 (0.28)	126 (0.68)		
Anxiety level (n, %)	Mild	8 (0.06)	32 (0.24)	57 (0.42)	38 (0.28)	80.378	<0.001
	Moderate	1 (0.01)	6 (0.06)	39 (0.36)	63 (0.58)		
	Severe	0 (0.0)	1 (0.01)	12 (0.14)	70 (0.84)		

Table 1 categorizes the severity of OCS as Healthy, Mild, Moderate, or Severe. Among the participants, 171 adolescents (52.3%) exhibited severe OCS, while 108 (35.9%) and 39 (12.9%) showed moderate and mild OCS, respectively, with only 9 adolescents (2.8%) classified as healthy.

### 3.2 Gender Differences

Females were significantly more likely to have severe OCS (147 out of 171) compared to males (24 out of 171), with a χ^2^ value of 9.287 and a *p*-value of 0.026, indicating a significant difference in OCS severity between sexes. Many severe OCS cases were observed in mid-adolescents aged 14–17 years (128 out of 171), though no significant age-related differences were found (χ^2^ = 7.594, *p* = 0.264).

### 3.3 Prediction of Depression and Anxiety on OCS

**Fig. 1.**
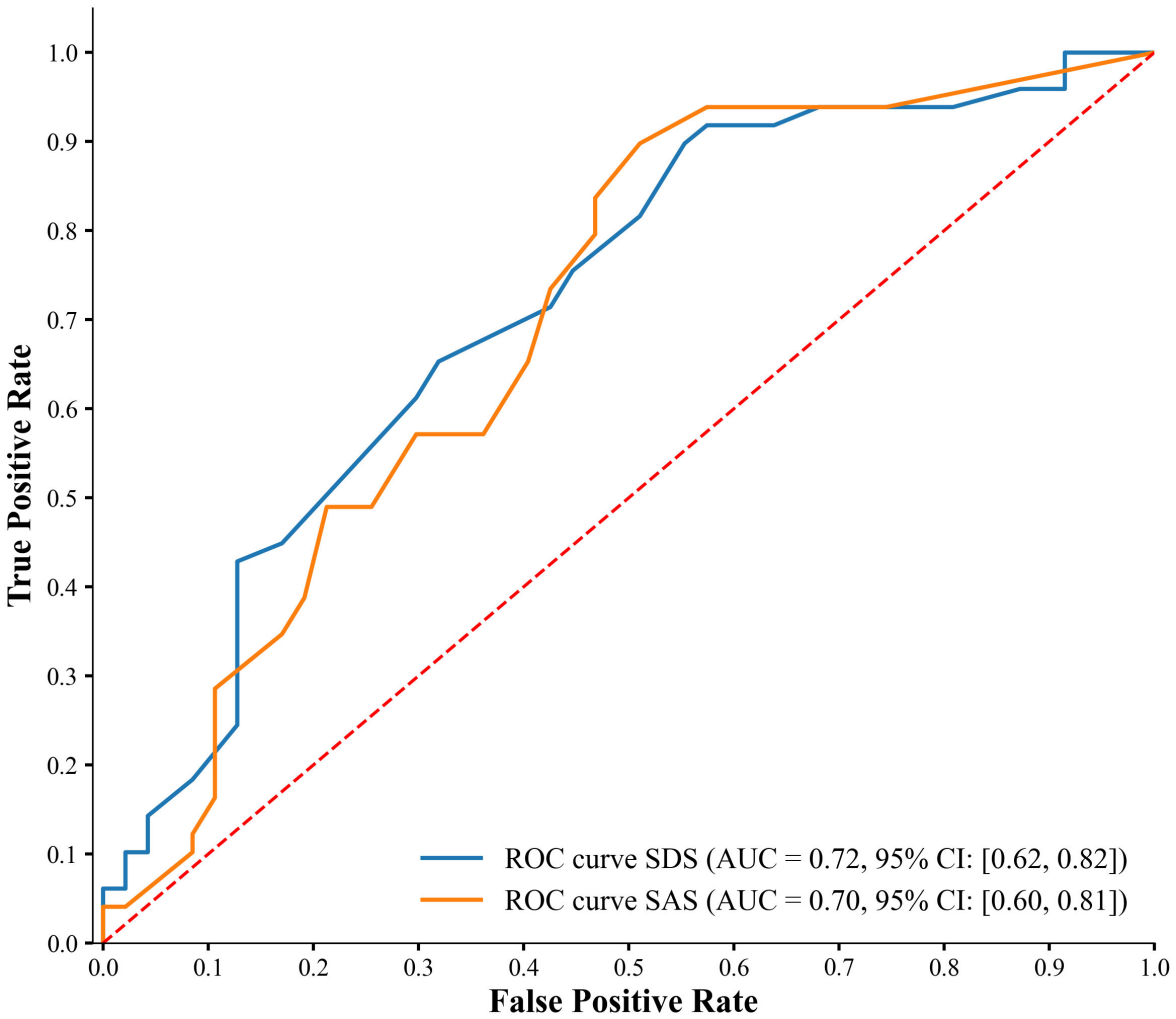
**ROC curve analysis for predicting OCS with depression and anxiety levels**.

After adjusting anxiety level and baseline characteristic (age and gender), depression level showed a statistically significant association with the presence of OCS (β = 0.073, Wald χ^2^ < 0.001, *p* < 0.005). This finding indicates that as depression levels increase, the odds of experiencing OCS also increase by a factor of 1.076 (Table 2). Similarly, after adjusting depression level and baseline characteristic (age and gender), the variable Self-Rating Anxiety Scale (SAS) demonstrated a statistically significant relationship with OCS (β = 0.066, Wald χ^2^ < 0.005, *p* < 0.001), implying that for each one-unit increase in anxiety level, the odds of having OCS increase by a factor of 1.069 (Table 2). This result is further used by the receiver operating characteristic (ROC) graph (Fig. 1).

**Table 2.** **Adjusted model for the prediction of depression and anxiety on obsessive compulsive symptom**.

	β	Wald χ^2^	*p*	*OR* ^3^	*95% CI* ^4^
SDS^1^	0.073	0.001	<0.005	1.076	1.036–1.117
SAS^2^	0.066	0.005	<0.001	1.069	1.029–1.110

Note. ^1^This model was adjusted for SAS, age, and gender. Self-Rating Depression Scale (SDS). ^2^This model was adjusted for SDS, age, and gender. Self-Rating Anxiety Scale (SAS). ^3^Odds Ratio. ^4^95% Confidence Interval.

The ROC curve analysis for predicting OCS using depression and anxiety levels revealed fair predictive abilities for both measures. The area under the curve (AUC) for the SDS (depression) curve was 0.72 (*95% confidence interval (CI)*: [0.62, 0.82]), indicating a moderate level of discrimination in predicting OCS based on depression levels. Similarly, the AUC for the SAS (anxiety) curve was 0.70 (*95% CI*: [0.60, 0.81]), showing a comparable level of discrimination for predicting OCS based on anxiety levels.

Comparing the two predictors, the depression levels (SDS) showed a slightly higher AUC than the anxiety levels (SAS), suggesting marginally better predictive power. However, the overlapping confidence intervals indicate no substantial difference in their predictive abilities.

## 4. Discussion

The findings of this study suggest that more than half of the adolescents with depressive and anxiety symptoms might experience severe obsessive-compulsive symptoms. Moreover, a significant correlation was found between depression and anxiety levels and the presence of OCS in adolescent patients. This indicates a strong predictive ability of both depression and anxiety level in predicting OCS. Although it was also found that female adolescents were much more likely to have severe OCS compared to males, the validity of this result is uncertain due to the significantly unbalanced male-to-female ratio.

The result of this study addresses a critical gap in the existing literature by exploring the predictive relationship in the opposite direction of that examined by several previous literature such as Hofmeijer-Sevink *et al*. (2018) [13]. While their research demonstrated that OCS are strongly associated with anxiety and depressive disorders and may predict their prevalence and severity, our findings provide evidence that depression and anxiety level can also independently predict the presence of OCS in adolescents. By examining this reverse direction, our study complements the findings of Hofmeijer-Sevink *et al*. (2018) [13] and adds to the understanding of the bidirectional nature of these comorbidities.

The bidirectional nature of these comorbidities could be supported by the hypothesis proposed by Goodwin (2015) [12], which suggests that shared genes and brain mechanisms contribute to the overlap between depression symptoms, anxiety symptoms, and OCS. The increased likelihood of comorbidity may be attributed to shared genetic traits or potential structural brain abnormalities in regions like the amygdala, which influence learned and behavioral responses to stimuli [9]. Another plausible explanation is that OCD represents a distinct underlying manifestation of anxiety [7]. As argued by Welkowitz *et al*. (2000) [10], OCS tends to increase in the presence of comorbid anxiety, and a substantial proportion of OCD patients also experience heightened general nervousness and worry [7].

However, there are some limitations to the present study. For instance, the unbalanced sample size may decrease the validity of the results. With a disproportionately higher number of female participants compared to male participants, the statistical power of the study is reduced. Moreover, the use of self-reports may compromise the validity of the study due to demand characteristics and social desirability bias. Moreover, although SDS, SAS, and SCL-90 have high reliability and validity in symptom assessment, they cannot replace professional clinical diagnoses. Therefore, future research should consider incorporating professional clinical evaluations for a more comprehensive understanding of the relationship between these mental health conditions.

Although there are several limitations, the present study is still important as it is the first to support the predictive ability of depressive and anxiety symptoms on the presence of OCS in adolescents. This may provide important insights into the mechanisms underlying the development of OCS and OCD during adolescence and contribute to early identification and intervention in this population. Furthermore, it also contributes to the understanding of the bidirectional nature of these comorbidities (depression, anxiety, OCD), which is essential for understanding and managing comorbid conditions and, therefore, for developing effective, tailored treatment strategies.

## 5. Conclusions

In conclusion, this study suggests that first-episode, drug-naive adolescent patients with comorbid depressive and anxiety symptoms are likely to also exhibit symptoms of OCD. The findings provide valuable insights into the predictive ability of depression and anxiety level in the development of OCS and OCD during adolescence, highlighting the importance of early identification and intervention. Additionally, a better understanding of the bidirectional nature of these comorbidities is essential for developing effective, tailored treatment strategies. Future studies should include a larger and more diverse sample, with the incorporation of professional clinical evaluations to further verify these results.

## Availability of Data and Materials

The data underpinning the results of this study are available by contacting the corresponding author, subject to a reasonable request.

## References

[1] American Psychiatric Association (2013). *Diagnostic and Statistical Manual of Mental Disorders*.

[2] Manfro PH, Pereira RB, Rosa M, Cogo-Moreira H, Fisher HL, Kohrt BA (2023). Adolescent depression beyond DSM definition: a network analysis. *European Child & Adolescent Psychiatry*.

[3] Shorey S, Ng ED, Wong CHJ (2022). Global prevalence of depression and elevated depressive symptoms among adolescents: A systematic review and meta-analysis. *The British Journal of Clinical Psychology*.

[4] Goldman LS, Nielsen NH, Champion HC (1999). Awareness, diagnosis, and treatment of depression. *Journal of General Internal Medicine*.

[5] Hazell P (2003). Depression in children and adolescents. *Evidence-based Mental Health*.

[6] Şentürk E, Geniş B, Coşar B (2021). Social Media Addiction in Young Adult Patients with Anxiety Disorders and Depression. *Alpha Psychiatry*.

[7] Tiller JWG (2013). Depression and anxiety. *The Medical Journal of Australia*.

[8] Sharma P, Rosário MC, Ferrão YA, Albertella L, Miguel EC, Fontenelle LF (2021). The impact of generalized anxiety disorder in obsessive-compulsive disorder patients. *Psychiatry Research*.

[9] Masellis M, Rector NA, Richter MA (2003). Quality of life in OCD: differential impact of obsessions, compulsions, and depression comorbidity. *Canadian Journal of Psychiatry. Revue Canadienne De Psychiatrie*.

[10] Welkowitz LA, Struening EL, Pittman J, Guardino M, Welkowitz J (2000). Obsessive-compulsive disorder and comorbid anxiety problems in a national anxiety screening sample. *Journal of Anxiety Disorders*.

[11] Huppert JD, Simpson HB, Nissenson KJ, Liebowitz MR, Foa EB (2009). Quality of life and functional impairment in obsessive-compulsive disorder: a comparison of patients with and without comorbidity, patients in remission, and healthy controls. *Depression and Anxiety*.

[12] Goodwin GM (2015). The overlap between anxiety, depression, and obsessive-compulsive disorder. *Dialogues in Clinical Neuroscience*.

[13] Hofmeijer-Sevink MK, Batelaan NM, van Megen HJGM, van den Hout MA, Penninx BW, van Balkom AJLM (2018). Presence and Predictive Value of Obsessive-Compulsive Symptoms in Anxiety and Depressive Disorders. *Canadian Journal of Psychiatry. Revue Canadienne De Psychiatrie*.

[14] ZUNG WW (1965). A SELF-RATING DEPRESSION SCALE. *Archives of General Psychiatry*.

[15] Zung WW (1971). A rating instrument for anxiety disorders. *Psychosomatics*.

[16] Wang J, Li Y, He E (1999). Reliability, and validity test of SCL-90 for middle school students and establishment of norm. *Chinese Mental Health Journal*.

